# Using Body Composition Analysis for Improved Nutritional Intervention in Septic Patients: A Prospective Interventional Study

**DOI:** 10.3390/nu15173814

**Published:** 2023-08-31

**Authors:** Kai-Yin Hung, Tzu-Hsiu Chen, Ya-Fen Lee, Wen-Feng Fang

**Affiliations:** 1 Division of Pulmonary and Critical Care Medicine, Department of Internal Medicine, Kaohsiung Chang Gung Memorial Hospital, Chang Gung University College of Medicine, Kaohsiung 83301, Taiwan; redrosahung@yahoo.com.tw; 2 Department of Nutritional Therapy, Kaohsiung Chang Gung Memorial Hospital, Kaohsiung 83301, Taiwankelly1973@cgmh.org.tw (Y.-F.L.); 3 Department of Nursing, Mei Ho University, Pingtung 91202, Taiwan; 4 Department of Respiratory Therapy, Kaohsiung Chang Gung Memorial Hospital, Chang Gung University College of Medicine, Kaohsiung 83301, Taiwan; 5 Department of Respiratory Care, Chang Gung University of Science and Technology, Chiayi 61363, Taiwan

**Keywords:** sepsis, body composition, phase angle

## Abstract

The study aimed to determine whether using body composition data acquired through bio-electrical impedance analysis (BIA) to adjust diet formulas could improve outcomes in septic patients. There were 132 septic patients in medical intensive care units enrolled in the prospective, randomized, double-blind, interventional study. For the intervention group, dietitians had access to BIA data for adjusting diet formulas according to body composition variables on days 1, 3, and 8. The patients were also stratified based on nutritional risk using the modified Nutrition Risk in Critically ill (mNUTRIC) score. Patients with intervention were more likely to achieve caloric and protein intake goals compared to the control group, especially in the low-risk group. The intervention did not significantly affect mortality, but the survival curves suggested potential benefits. The high-risk group had longer ICU stays and mechanical ventilation duration, which were mitigated by the intervention. Certain body composition variables (e.g., extracellular water to total body water ratio and phase angle) showed differences between high-risk and low-risk groups and may be related to patient outcomes. Non-invasive body composition assessment using BIA can help dietitians adjust diet formulas for critically ill septic patients. Body composition variables may be associated with sepsis outcomes, but further research with larger patient numbers is needed to confirm these findings.

## 1. Introduction

Sepsis, a life-threatening systemic illness characterized by organ dysfunction resulting from infection, is a common reason for admission to intensive care units (ICUs) and is associated with high mortality rates [1]. The key indicator of sepsis-induced organ dysfunction is an acute change in the total sequential organ failure assessment (SOFA) score [2,3]. However, this score does not account for nutritional status or body composition variables. Our previous data revealed that patients with immune deficiencies or impaired immune responses had poorer outcomes [4], especially those with underlying comorbidities [5]. In ICU patients with sepsis, poor nutritional status and immune response can further worsen the condition. Notably, patients who did not receive sufficient calories by day 7 had higher 28-day mortality rates [6].

Hospitalized patients are often at risk of malnutrition, which may be due to disease-related catabolic states or inadequate nutrition supply [7,8,9,10]. Early enteral feeding has been proven to reduce ICU and hospital mortality and has become a current standard practice [11]. Conversely, some studies have suggested that intentional hypocaloric feeding could reduce infection rates and mortality, irrespective of nutritional risk [12,13,14]. The conflicting associations regarding true caloric needs and clinical outcomes may reflect the complex and heterogeneous nature of the ICU population. Furthermore, these studies did not consider body composition variables when adjusting the diet formula based on the patients’ body composition. 

Body composition refers to the percentage of fat, bone, and muscle in a person’s body. Non-invasive bio-electrical impedance analysis (BIA) can be used to analyze body composition, allowing for the assessment of different weight components such as lean body mass and fat mass. This method is based on the electrical properties of tissues and reflects the relationship between water content and the body’s electrical resistance. Different body tissues have different water content, electrical conductivity, and electrical resistance. A change in these parameters in individual organs and tissues indicates the presence of pathology. We used these changes in sepsis to help the dietitian. By monitoring body composition variables for muscle loss, fluid levels, and cellular health, we may gain valuable insights into sepsis outcomes. In particular, lean analysis enables us to monitor lean body mass and extracellular water (ECW)/total body water (TBW) values in the body. Additionally, phase angle (PA), which measures cellular resistivity and reflects cell membrane integrity and overall cellular health, could serve as a prognostic factor of survival [15,16]. BIA has been proposed as a valuable tool for assessing nutritional risk and monitoring the response to nutrition in critical care settings in the future [17]. 

In our study, we defined nutritional intervention as the dietitian’s ability to utilize the patient’s BIA data as an additional reference for prescribing the appropriate diet formula. The primary aim of this study was to determine whether septic patients in the nutritional intervention group experienced better outcomes ([1] primary outcomes: length of stay and mortality, [2] secondary outcomes: amount of caloric and protein intake, percentage of patients meeting caloric and protein goals) compared to septic patients in the control (non-interventional) group. The dietitian in charge of the patients in the nutritional intervention group was provided with the BIA data, whereas the control group did not receive such intervention. Additionally, patients were stratified by nutritional risk according to the modified Nutrition Risk in Critically Ill (mNUTRIC) score for further analysis.

## 2. Materials and Methods

### 2.1. Patient Enrollment

We enrolled septic patients from medical ICUs at Kaohsiung Chang Gung Memorial Hospital, a 2700-bed tertiary teaching hospital in southern Taiwan, between August 2020 to December 2022 (ClinicalTrials.gov Identifier: NCT04989569). Patients were excluded if they met any of the following criteria: (1) age < 18 years, (2) conditions that may interfere with the accuracy of BIA measurement (e.g., limb amputation, skin wounds in areas for sensor attachment), and (3) absence of informed consent signed by patients or their surrogates.

### 2.2. Study Design

This is a prospective, randomized, double-blind, interventional study. The investigators used a random number generator to divide the enrolled patients into two groups: the control group (dietitian-prescribed diet formula based on clinical judgment without knowledge of BIA data) and the intervention group (BIA data disclosed to the dietitian for reference). The registered dietitians in charge of the patients in the intervention group were encouraged to use BIA data for adjusting the diet formula according to the patient’s body composition variables. Patients and other medical team members caring for the enrolled patients were blinded to the randomization. The study design was approved by the Institutional Review Board of Chang Gung Memorial Hospital without any conflict of interest (Approval No: 201901875A3C501).

### 2.3. Diet Formula Adjustment Policy

The nutritionist creates a diet based not only on the calorie and protein principle. There are other components in the food besides those mentioned, as well as carbohydrates, fat, sodium, potassium, calcium, and phosphorus content. We also take enteral and parenteral nutrition into consideration. The dietitians recommended the amounts of caloric and protein intakes based on the guidelines for adult critically ill patients, which were endorsed by the Society of Critical Care Medicine (SCCM) and the American Society for Parenteral and Enteral Nutrition (A.S.P.E.N.) [18]. Registered dietitians provided and adjusted nutritional prescriptions for all participants primarily based on clinical conditions, laboratory data results, and discussions with the medical team. In the intervention group, the additional policy adopted by the dietary treatment guidelines allowed for adjusting the dietary concentration when there was excessive extracellular water, as long as gastrointestinal tolerance was considered. If skeletal muscle mass loss occurred rapidly, the dietitians could adjust the diet formula to provide higher protein. For patients with acute renal failure and excessive extracellular water accumulation and muscle loss, the dietitians would adjust the concentration of protein, sodium, potassium, calcium, and phosphorus content in the formula.

### 2.4. Measurements

All enrolled patients underwent non-invasive body composition measurements on day 1, day 3, and day 8 (if alive). The data for the SOFA score were also collected on day 1, day 3, and day 8. Dietitians recorded daily actual energy (caloric) and protein intake of the patients, including intake from enteral and parenteral routes, from day 1 to day 8. The goals for caloric intake were set at 20 kcal/kg and for protein intake at 1.2 g/kg per day.

### 2.5. BIA Measurements

We performed the measurement of body composition using the InBody S10 (Biospace Co, Ltd., Cheonan, Republic of Korea) according to the manufacturer’s protocol. This machine is a body composition analyzer based on the direct segmental multi-frequency bioelectrical impedance method. In brief, the examinee assumed a supine posture for approximately 10–15 min before the test, ensuring no contact between the examinee’s body and conductors during testing. We used touch-type electrodes, attaching them to the examinee’s hands and feet. Patients’ latest weight and height were entered as essential information for body composition analysis. The InBody S10 then analyzed the measurement results based on the input data of the patient. The outputs included intracellular water, extracellular water, total body water (TBW), protein mineral, body fat, soft lean mass, fat-free mass (FFM), weight, skeletal muscle mass, body fat mass, percentage of body fat, body mass index (BMI), segmental lean analysis, segmental water analysis, total and segmental water ratio (ECW/TBW), body cell mass (BCM), bone mineral content (BMC), arm circumference (AC), arm muscle circumference (AMC), waist circumference, visceral fat area, basal metabolic rate (BMR), TBW/FFM, body water history (12 times accumulated results), and impedance of each segment and frequencies (impedance, reactance, phase angle).

### 2.6. Statistics

All data were presented as mean and standard deviation (SD) or median and interquartile range (IQR) depending on normal distribution. The Pearson chi-square test was used for categorical data, and Student’s *t*-test or Mann–Whitney U test was used for numerical data to compare between groups. For comparative analyses among four groups (2 × 2; combined intervention or control with high or low nutritional risk score [modified NUTRIC score]), we used the Pearson chi-square and one-way analysis of variance (ANOVA) or Kruskal–Wallis test as a non-parametric alternative to ANOVA. Cox regression model for survival analysis and Kaplan–Meier analysis were used to determine the effect of the groups on patient survival. We compared mortality hazard ratios between groups using Cox proportional-hazards regression analysis for 28-day mortality. Statistical analyses were conducted with a two-tailed *p*-value < 0.05 considered as significant. SPSS software (version 22; IBM Corp., Armonk, NY, USA) was used for all statistical analyses.

## 3. Results

### 3.1. Patients Grouped by Intervention or Nutritional Risk

#### 3.1.1. Baseline Characteristics

**Figure 1:**
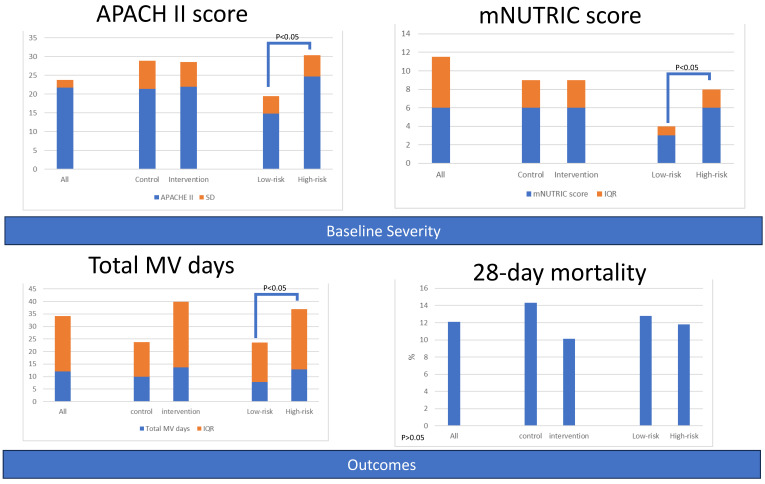
Baseline severity and outcomes of patients, grouped by intervention or nutritional risk.

A total of 132 patients (63 in the control group, 69 in the intervention group) were enrolled in the study. The baseline characteristics, severity scores, suspected site of infection, and underlying comorbidities were comparable between the two groups. Additionally, patients were categorized into high-risk (*n* = 93) and low-risk (*n* = 39) groups based on their mNUTRIC scores (≥6 vs. ≤5). Patients in the high-risk group were older and had higher severity scores (APACHE II, PSI, CURB 65) and comorbidities (Charlson comorbidity index, hypertension, diabetes mellitus, chronic kidney disease). The lung was the most commonly suspected source of infection causing sepsis in the high-risk group (Table 1 and Figure 1).

#### 3.1.2. Primary Outcomes

**Figure 2:**
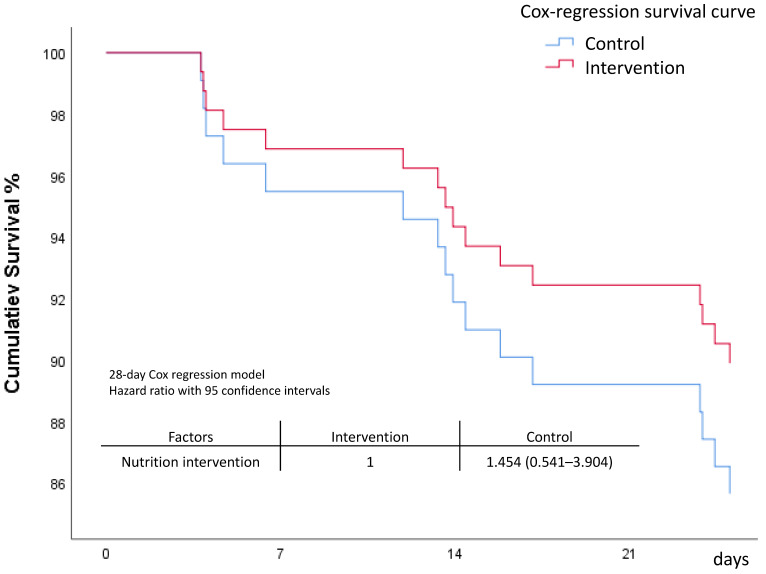
Survival comparison between intervention and control groups.

**Figure 3:**
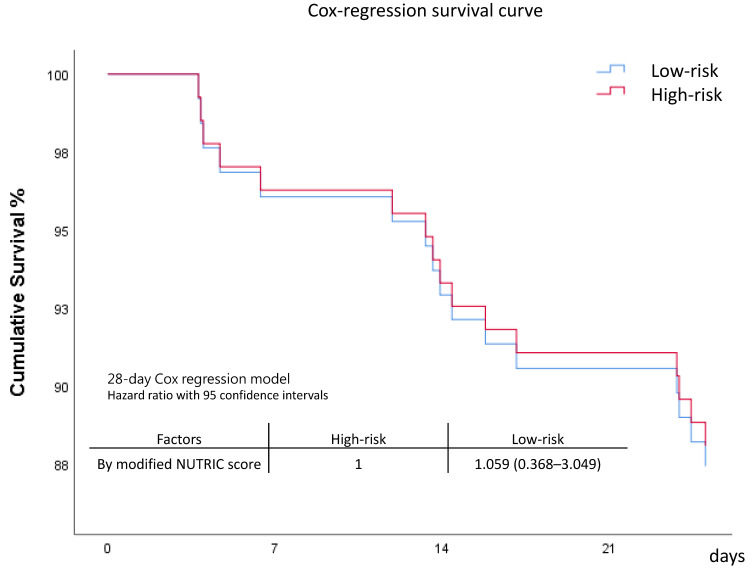
Survival comparison between high-risk and low-risk groups.

Comparison between the control group and intervention group revealed comparable outcomes. However, the intervention group seemed to have lower day 7 and day 28 mortality rates, although the difference was not statistically significant. Patients in the high-risk group had a longer length of stay in the ICU and on mechanical ventilator compared to the low-risk group. Mortality rates between the two risk groups were comparable (Table 2 and Figure 1). The survival curve of the intervention group appeared to be better, but the survival curves between the control and intervention groups did not show significant separation (Figure 2 and Figure S1). The patients with higher modified NUTRIC scores appeared to have poorer survival, but the survival curves did not show significant separation (Figure 3 and Figure S2).

#### 3.1.3. Secondary Outcomes: Amounts of Caloric and Protein Intake

**Figure 4:**
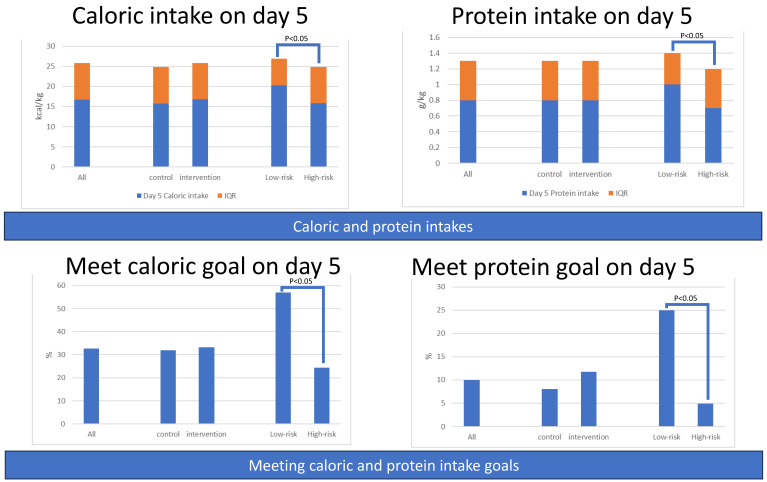
Amounts of caloric and protein intake of patients and percentage of patients meeting caloric or protein goals, grouped by intervention or nutritional risk.

The amounts of caloric intake and protein intake during the first week were comparable between the control and intervention groups. However, the amounts of caloric intake and protein intake were significantly higher in the low-risk group compared to the high-risk group on most days of the first week (Figure 4 and Table S1).

#### 3.1.4. Secondary Outcomes: Percentage of Patients Meeting Caloric or Protein Goals

There was no difference between the control and intervention groups regarding the percentage of patients who met the caloric goal (20 kcal/kg) and protein goal (1.2 g/kg) during the first week. However, compared with the high-risk group, patients in the low-risk group had a higher percentage of patients who met the caloric and protein goals during the first week (Figure 4 and Table S2).

#### 3.1.5. Serial Severity Score for All Patients and Grouped by Intervention or Risk

**Figure 5:**
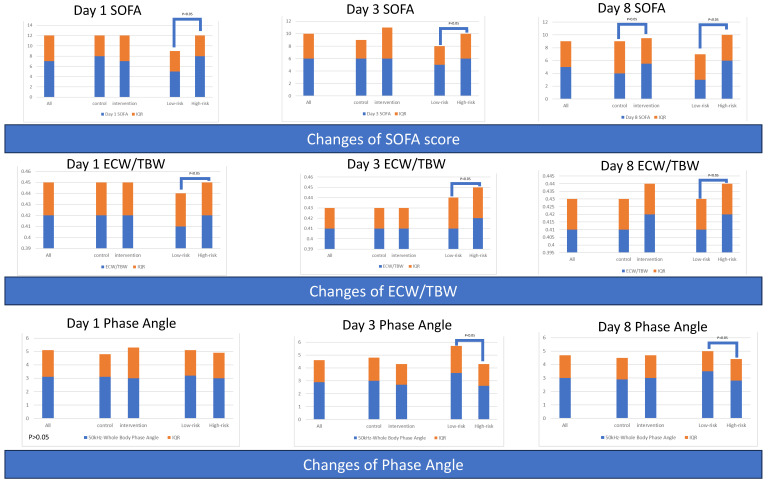
Serial severity scores and serial body composition variables of patients, grouped by intervention or nutritional risk.

The baseline SOFA scores were comparable between the control and intervention groups, but the SOFA score was higher in the intervention group on day 8. The low-risk group had better SOFA scores at baseline and during the first week compared to the high-risk group. However, the decline of SOFA from day 1 to day 3 was more significant in the high-risk group (Table 3 and Figure 5).

#### 3.1.6. Serial Body Composition Variables for All Patients and Grouped by Intervention or Risk

Serial values regarding body composition variables were comparable between the control and intervention groups. The high-risk group had higher serial ratios of extracellular water to total body water (ECW/TBW). The low-risk group had borderline higher values of phase angle (50 kHz-Whole Body Phase Angle) at baseline and significantly higher phase angle values compared to the high-risk group. Moreover, an increase in body fat mass was borderline higher in the high-risk group (Table 4 and Figure 5).

### 3.2. Grouped by Intervention and Risk

#### 3.2.1. Baseline Characteristics of Four Groups

Patients were divided into four subgroups based on their mNUTRIC scores and whether they received nutritional intervention. Group A comprised patients with high mNUTRIC scores who received nutritional intervention, Group B included patients with low mNUTRIC scores who received nutritional intervention, Group C consisted of patients with high mNUTRIC scores who did not receive nutritional intervention, and Group D contained patients with low mNUTRIC scores who did not receive nutritional intervention. The demographic characteristics of these subgroups are shown in Table 5.

#### 3.2.2. Primary Outcomes: Length of Stay and Mortality among Four Groups

**Figure 6:**
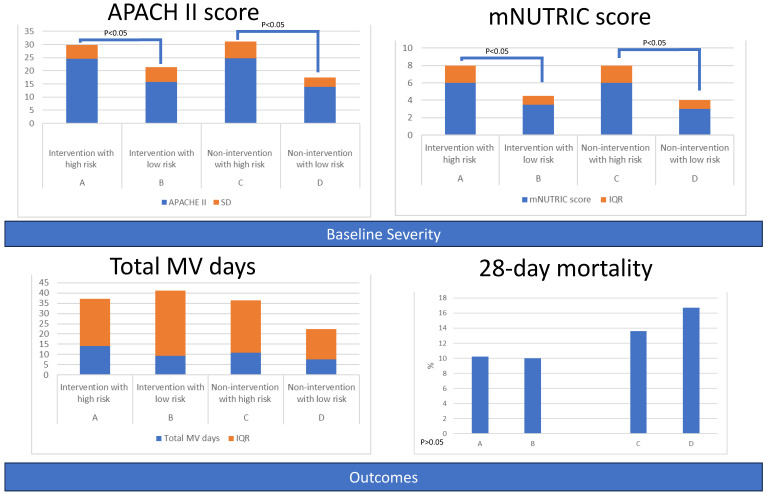
Baseline severity and outcomes of patients, grouped by intervention and nutritional risk.

**Figure 7:**
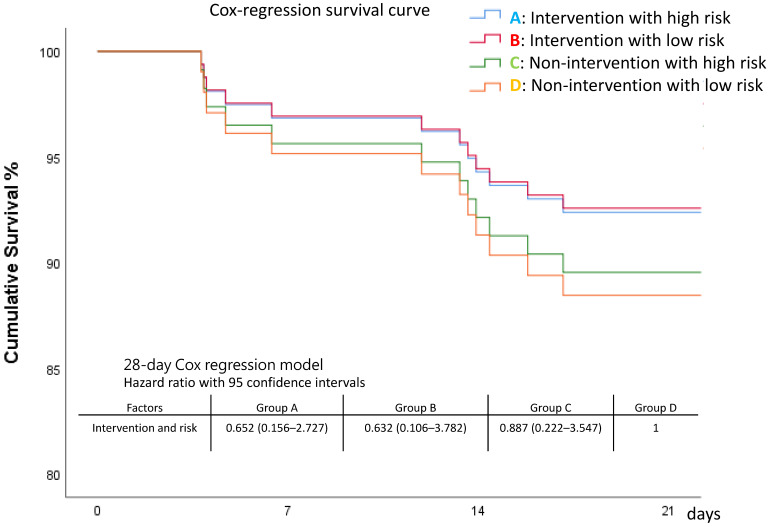
Survival among the four groups.

ICU, 7-day, and 28-day mortality rates appeared lowest in Group B (intervention with low risk), although without statistical significance. Patients at high risk had a longer length of stay on mechanical ventilation (Table 5 and Figure 6). The survival curves also showed that Curve B appeared better, followed by Curve A, Curve C, and Curve D, but the differences were not statistically significant (Figure 7 and Figure S3, Table S3).

#### 3.2.3. Secondary Outcomes: Amount of Caloric and Protein Intake among Four Groups

**Figure 8:**
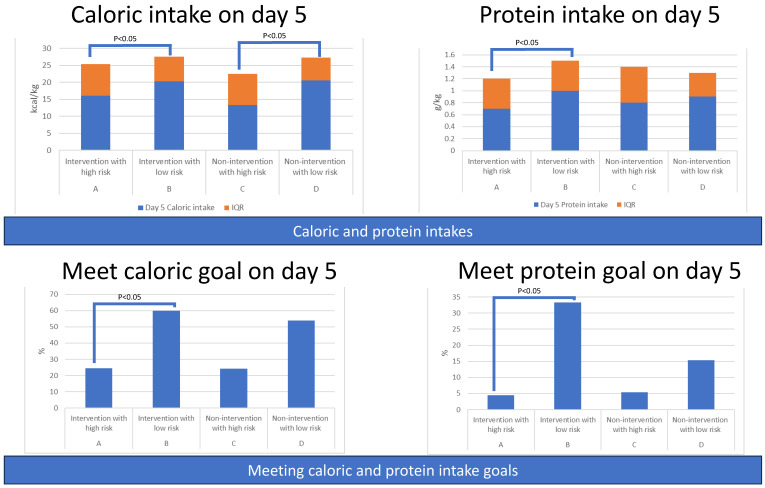
Amounts of caloric and protein intake of patients and percentage of patients meeting caloric or protein goals, grouped by intervention and nutritional risk.

Patients with low risk (Group B and Group D) had higher amounts of caloric and protein intake from day 1 to day 8 compared to corresponding patients with high risk (Group B vs. Group A; D vs. C). Statistical significance was noted during most of the days in the intervention groups (A vs. B) rather than the non-intervention groups (C vs. D), indicating that intervention played a significant role (Table S4 and Figure 8).

#### 3.2.4. Secondary Outcomes: Percentage of Patients Meeting Caloric or Protein Goals among Four Groups

Patients in Group B (Intervention with low risk) had a higher percentage of achieving the daily caloric goal (20 kcal/kg) than the other three groups on day 3, day 4, and day 5. Similar results were observed for the proportion of patients meeting the protein goal (1.2 g/kg), with Group B having the highest percentage (Table S5 and Figure 8).

#### 3.2.5. Serial Severity Scores among Four Groups

**Figure 9:**
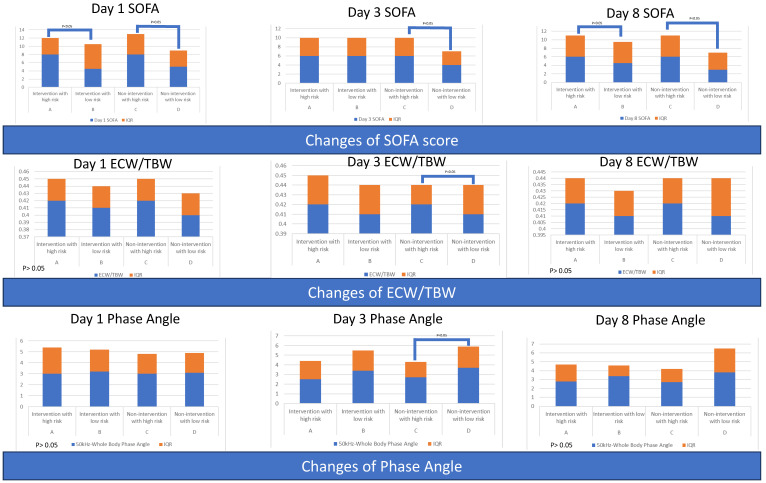
Serial severity scores and serial body composition variables of patients, grouped by intervention and nutritional risk.

The serial sepsis severity scores are shown in Table S6 and Figure 9. Group D (non-intervention with low risk) had the lowest serial SOFA score among the groups.

#### 3.2.6. Serial Body Composition Variables among Four Groups

Patients in Group C (non-intervention with high risk) had a borderline lowest phase angle (related to poor cellular health) on day 8, although the phase angle in Group D was comparable. The change in phase angle from day 1 to day 3 between these two groups was significant, indicating that Group C experienced consistent worsening of cellular health. Group B had preserved more soft lean mass, skeletal muscle mass, and higher phase angle (related to good cellular health) (Table S7 and Figure 9).

### 3.3. Items of Statistically Significant Differences

The items of statistically significant differences between groups and among groups are listed below (Table 6 and Table 7).

## 4. Discussion

In this prospective, randomized, controlled study, we have obtained valuable findings. Firstly, patients who received intervention were more likely to achieve higher caloric and protein intake, particularly those in the low-risk group compared to the high-risk group. Secondly, critically ill patients with high nutrition risk on admission (high-risk group) showed longer lengths of stay in the ICU and a higher need for mechanical ventilation. However, in the intervention group, the differences in ICU stay and duration of mechanical ventilation between high-risk and low-risk groups were mitigated. Thirdly, although not statistically significant, the survival curves appeared more favorable in the intervention group compared to the control group and in the low-risk group compared to the high-risk group. Fourthly, non-invasive body composition variables, such as ECW/TBW and 50 kHz-whole body phase angle, could assist dietitians in adjusting diet formulas and may be associated with the outcomes of critically ill patients with sepsis. 

In a state of sepsis, the so-called oxidative stress is due to inflammation and the generation of a huge amount of free radicals that damage cell membranes and make them particularly permeable. If the food contains some of the natural antioxidants-polyphenols, flavonoids, vitamins, etc., they may contribute to the control of inflammation. Such data would allow a broader view of the pathogenesis of sepsis. However, it is out of the scope of the current study. Nevertheless, managing the outcomes of sepsis requires a multi-pronged approach that looks at different aspects of the patient’s health and response to treatment.

The additional procedure in this study involved non-invasive measurement of body composition variables three times (on day 1, 3, and 8) for all enrolled patients. The procedure took about 10 min each time and did not interfere with patient care. In the control group, BIA data was not disclosed to the clinical team, while in the intervention group, the data were released to the registered dietitian for possible adjustments in the diet formula. The study was designed to ensure blinding of other medical staff in the ICU. The presence of a dietitian in the ICU can be crucial for optimal patient care, but it is worth noting that not all ICUs have access to a dietitian [19]. For those without a dietitian, the supplemental BIA data could serve as valuable additional information for the medical teams. Moreover, the time required for body composition measurement is not too long, making it suitable for a high-stress ICU setting with high levels of burnout [20].

The stratification of nutritional risk by mNUTRIC score upon admission to the ICU proved to be effective. It outperformed body mass index in defining nutritional status and could be useful for predicting survival in patients with sepsis. The modified NUTRIC score includes age, Acute Physiology and Chronic Health Evaluation (APACHE II score), SOFA score, number of comorbidities, and hospital stay before admission to ICU. It has also shown promise as a useful tool for predicting mortality in severe pneumonia [21], which is the most common source of infection in ICU patients with sepsis. Therefore, including severity scores for pneumonia (PSI and CURB-65 score) at baseline provided a reference point for comparison.

In our study, we not only collected baseline characteristics of enrolled patients but also provided serial SOFA scores and body composition variables for comparison both between and among groups. These data were relevant to understanding sepsis progression and changes in nutritional status. For instance, a sequential decrease in SOFA score indicated an improvement in sepsis. We hypothesized that dynamic changes in data would be more informative than one-time measurements. This hypothesis was supported by our previous research, which demonstrated the importance of dynamic data for predicting outcomes in septic patients [22].

To assess secondary outcomes, we comprehensively analyzed the caloric and protein intake amounts and the percentage of patients meeting preset dietary goals from day 1 to day 8. Our findings revealed that intervention seemed to facilitate patients in the low-risk group to achieve their energy targets compared to those in the high-risk group. This difference could be attributed to the high-risk group’s greater illness severity, which may have affected their ability to digest and have slower gastric motility. Additionally, the higher prevalence of underlying morbidities such as diabetes mellitus (DM) and chronic kidney disease (CKD) in the high-risk group may have exacerbated the condition. Stress-induced upper gastrointestinal bleeding, which was common in severe septic patients [23], might have further hindered scheduled nasogastric tube feeding in these patients. Although the intervention of involving a dietitian did not significantly improve diet input for high-risk patients based on body composition variables, it did seem to enhance diet adjustment for patients in the low-risk group, who appeared to tolerate changes better.

Regarding mortality, we observed that early changes in SOFA scores could serve as a prognostic marker for 28-day sepsis mortality [24]. After treatment, serial SOFA scores demonstrated a decline in both intervention and control groups as well as in the high-risk and low-risk categories. However, on day 8, the intervention group exhibited higher SOFA scores compared to the control group, indicating a less favorable recovery. Notably, the decline in SOFA score from day 1 to day 3 was more evident in the high-risk group than in the low-risk group, suggesting a faster recovery rate in the former. These unexpected features may partially explain why the survival curves, although appearing better in the intervention group compared to the control, or in the low-risk group compared to the high-risk group, did not reach statistical significance. Our simple intervention did not significantly impact mortality. It is essential to acknowledge that septic patients’ survival depends on multiple factors, including immune response, and not solely on caloric intake [4,6,17]. It is crucial to emphasize that our intervention did not harm septic patients, indicating its safety. However, it is evident that addressing sepsis outcomes requires a multifaceted approach that considers various aspects of patient health and response to treatment.

An essential aspect of our study was the collection of serial body composition variable data, which hold potential for monitoring nutritional status. The nutritional status of patients is closely linked to their exercise capacity, which, in turn, may be associated with the duration of mechanical ventilation. Building on insights from our previous study, where appropriate diet intervention was shown to alter nutritional status [21], we aimed to investigate whether our intervention could also influence outcomes in this regard. Each body composition variable we examined could carry its own clinical significance. For instance, we observed that positive accumulated fluid balance, leading to body weight gain, was associated with unfavorable outcomes in severe influenza pneumonia [25]. The accumulation of fluid might be linked to increased Extracellular Water (ECW) to Total Body Water (TBW) ratio, which can be easily measured using Bioelectrical Impedance Analysis (BIA). We noted some significant differences in ECW/TBW and phase angle between the high-risk and low-risk groups. These differences could partly be attributed to variations in ICU stay duration and mechanical ventilation duration between the groups, though our study did not establish a definitive relationship. However, a prospective study, in which the majority of patients were admitted to the ICU due to acute respiratory failure rather than sepsis, found that critically ill patients with lower phase angles tended to have higher in-hospital mortality [26]. These body composition variables might also have implications for the survival of septic patients. For example, higher values of the phase angle were indicative of better cellular health and could potentially serve as a biomarker for sepsis resolution. Additionally, other variables such as soft lean mass and skeletal muscle mass may play a role in determining the duration of mechanical ventilation. Critically ill patients often experience rapid muscle wasting during the first 7 days [27], which correlates with the severity of their illness, length of hospital stay, and mortality risk [28]. Although we observed lower soft lean mass and skeletal muscle mass in patients at high-risk and receiving intervention (group A compared to group B), these differences did not reach statistical significance. A larger-scale study might be necessary to provide more robust conclusions in this regard.

The strength of our study lies in its prospective, randomized, double-blind, interventional design. Blinding was ensured for patients and most medical staff except for the dietitian. Comprehensive data on caloric and protein intake, including energy from intravenous infusions and sedation medication, were recorded, enhancing the reliability of the results. Moreover, the study included serial data on SOFA scores, body composition variables, and caloric and protein intakes throughout the study period, rather than relying solely on admission data. The intervention itself is relatively simple and feasible for application in clinical practice.

However, our study has some limitations. The number of enrolled patients was smaller than expected due to the COVID-19 pandemic, which led to difficulties in conducting relationship analysis. The quarantine policy prevented the enrollment of septic patients with COVID-19, limiting the generalizability of our results to this particular population. Additionally, our study was conducted in a tertiary hospital with adequate resources and a highly skilled medical team specializing in treating sepsis [29]. Therefore, the results may not be applicable to smaller hospitals with more limited resources. Nevertheless, the non-invasive procedure of body composition analysis is straightforward and can be easily incorporated into protocols in various settings.

In summary, using non-invasive body composition variables obtained through bio-electrical impedance analysis to adjust diet formulas is beneficial for critically ill septic patients. Patients who received this nutritional intervention were more likely to achieve higher caloric and protein intake, particularly in low-nutritional risk patients according to the modified nutrition risk in critically ill score. Although the intervention only showed borderline improved survival, it appeared to influence the length of stay in the ICU and the duration of mechanical ventilation, which tended to be longer in high-risk patients. Among the body composition variables, ECW/TBW was significantly higher in the high-risk group, while the phase angle was higher in the low-risk group.

## 5. Conclusions

Non-invasive body composition variables, such as ECW/TBW and phase angle, could assist dietitians in adjusting diet formulas and may be associated with improved outcomes in critically ill patients with sepsis.

## Figures and Tables

**Table 1:** Baseline characteristics of patients, grouped by intervention or nutritional risk.

	All	Control	Intervention		Low-Risk	High-Risk	
	N = 132	N = 63	N = 69	*p* †	N = 39	N = 93	*p* †
Demographic characteristics, mean (SD) or median (IQR)							
Age (years)	71.0 (14.7)	69.2 (11.9)	72.6 (12.0)	0.101	63.7 (10.0)	74.1 (11.5)	0.000
Gender(female), *n* (%)	57 (5.50)	28.0 (44.4)	29.0 (42.0)	0.780	14.0 (35.9)	43.0 (46.2)	0.327
Body weight (kg)	60.5 (7.0)	60.9 (15.0)	60.2 (14.5)	0.763	61.2 (14.9)	60.2 (14.7)	0.720
BMI (kg/m^2^)	23.3 (44.40)	23.2 (5.9)	23.3 (5.2)	0.932	22.9 (5.5)	23.4 (5.6)	0.619
APACHE II	21.8 (2.0)	21.4 (7.5)	22.0 (6.6)	0.636	14.8 (4.7)	24.7 (5.7)	0.000
PSI score	133.9 (3.0)	133.4 (47.7)	134.4 (41.6)	0.902	108.3 (31.9)	144.7 (44.7)	0.000
CURB-65 score	2.0 (3.0)	2.0 (1.0)	2.0 (2.0)	0.149	1.0 (1.0)	2.0 (2.0)	0.000
Charlson comorbidity index	5.0 (14.7)	5.0 (4.0)	5.0 (3.0)	0.804	4.0 (2.0)	6.0 (4.0)	0.000
mNUTRIC score	6.0 (5.5)	6 (3.0)	6.0 (3.0)	0.776	3.0 (1.0)	6.0 (2.0)	0.000
Site of suspected infection, *n* (%)							
Lung	109 (82.6)	53 (84.1)	56 (81.2)	0.655	27 (69.2)	82 (88.2)	0.009
Intra-abdomen	6 (4.5)	2 (3.2)	4 (5.8)	0.472	1 (2.6)	5 (5.4)	0.481
UTI	93 (70.5)	40. (63.5)	53 (76.8)	0.095	26 (66.7)	67 (72.0)	0.538
Bacteremia	71 (53.8)	31 (49.2)	40 (58.0)	0.315	23 (59.0)	48 (51.6)	0.441
Others	5 (3.8)	4 (6.3)	1 (1.4)	0.142	3 (7.7)	2 (2.2)	0.130
Comorbidities, *n* (%)							
Coronary artery disease,	23 (17.4)	11 (17.5)	12 (17.4)	0.992	4 (10.3)	19 (20.4)	0.161
Hypertension	76 (57.6)	36 (57.1)	40 (58.0)	0.924	17 (43.6)	59 (63.4)	0.036
COPD	15 (11.4)	9 (14.3)	6 (8.7)	0.314	3 (7.7)	12 (12.9)	0.391
Cancer	44 (33.3)	26 (41.3)	18 (26.1)	0.066	13 (33.3)	31 (33.3)	1.000
Liver cirrhosis	6 (4.5)	3 (4.8)	3 (4.3)	0.910	1 (2.6)	5 (5.4)	0.481
Diabetes mellitus	65 (49.2)	29 (46.0)	36 (52.2)	0.482	13 (33.3)	52 (55.9)	0.018
CKD	43 (32.6)	22 (34.9)	21 (30.4)	0.584	7 (17.9)	36 (38.7)	0.021

† Comparison analyses between two groups by Mann–Whitney U tests or chi-squared tests for categorical variables. Abbreviations: SD, standard deviation; IQR, interquartile range; BMI, body mass index; APACHE, acute physiology and chronic health evaluation; PSI, pneumonia severity index, CURB-65: Confusion, blood urea > 42.8 mg/dL, respiratory rate > 30/min, blood pressure < 90/60 mmHg, age > 65; mNUTRIC, modified nutrition risk in critically ill; UTI: urinary tract infection; COPD, chronic obstructive pulmonary disease; CKD, chronic kidney disease.

**Table 2:** Primary outcomes of patients, grouped by intervention or nutritional risk.

	All	Control	Intervention		Low-Risk	High-Risk	
	N = 132	N = 63	N = 69	*p* †	N = 39	N = 93	*p* †
Length of stay, median (IQR)							
ICU Days	9.0 (8.4)	7.8 (10.1)	10.0 (8.8)	0.101	6.6 (9.6)	10.0 (7.8)	0.024
Hospital days	26.6 (31.0)	24.4 (29.0)	29.0 (33.5)	0.390	21.6 (24.6)	29.6 (30.4)	0.176
Total MV days	12.0 (22.2)	9.9 (13.8)	13.7 (26.1)	0.073	7.9 (15.7)	12.9 (24.1)	0.021
mortality, *n* (%)							
ICU Mortality,	15 (11.4)	7 (11.1)	8 (11.6)	0.931	4 (10.3)	11 (11.8)	0.832
Hospital Mortality	27 (20.5)	13 (20.6)	14 (20.3)	0.961	7 (17.9)	20 (21.5)	0.693
7-day Mortality	5 (3.8)	4 (6.3)	1 (1.4)	0.142	1 (2.6)	4 (4.3)	0.652
28-day Mortality	16 (12.1)	9 (14.3)	7 (10.1)	0.468	5 (12.8)	11 (11.8)	0.834

† Comparison analyses between two groups by Mann–Whitney U tests or chi-squared tests for categorical variables. Abbreviations: IQR, interquartile range; ICU, intensive care unit; MV: mechanical ventilation; UTI, urinary tract infection.

**Table 3:** Serial severity scores of patients, grouped by intervention or nutritional risk.

	All	Control	Intervention		Low-Risk	High-Risk	
	N = 132	N = 63	N = 69	*p* †	N = 39	N = 93	*p* †
Serial severity scores, median (IQR)							
Day 1 SOFA	7 (5)	8 (4)	7 (5)	0.711	5 (4)	8 (4)	0.000
Day 3 SOFA	6 (4)	6 (3)	6 (5)	0.474	5 (3)	6 (4)	0.007
Day 8 SOFA	5 (4)	4 (5)	5.5 (4)	0.014	3 (4)	6 (4)	0.000
Difference of severity score (day 3 value minus day 1 value), median (IQR)							
ΔSOFA	−2 (3)	−2 (4)	−2.0 (4)	0.057	−1.5 (5)	−2 (3)	0.030

† Comparison analyses between two groups by Mann–Whitney U tests. abbreviations: IQR, interquartile range; SOFA, sequential organ failure assessment.

**Table 4:** Serial body composition variables of patients, grouped by intervention or nutritional risk.

	All	Control	Intervention		Low-Risk	High-Risk	
	N = 132	N = 63	N = 69	*p* †	N = 39	N = 93	*p* †
Day 1, median (IQR)							
Total Body Water (kg)	29.8 (8.7)	30.9 (8.9)	29.6 (8.7)	0.099	30.2 (7.9)	29.8 (8.2)	0.826
Intracellular Water (kg)	17.8 (5.0)	19.0 (5.3)	17.6 (5.2)	0.087	17.8 (4.9)	17.9 (5.0)	0.998
Extracellular Water (kg)	12.2 (3.8)	12.7 (4.2)	11.9 (3.6)	0.107	12.3 (3.6)	11.7 (4.0)	0.673
Body Fat Mass (kg)	17.2 (3.6)	15.6 (12.2)	19.1 (14.7)	0.089	16.6 (12.9)	17.9 (14.2)	0.965
Soft Lean Mass (kg)	38.4 (10.6)	39.6 (11.4)	37.6 (11.1)	0.082	38.4 (10.1)	38.4 (10.4)	0.850
Skeletal Muscle Mass (kg)	21.3 (6.5)	22.7 (7.0)	21.0 (6.8)	0.081	21.2 (6.4)	21.3 (6.4)	0.976
ECW/TBW	0.42 (0.03)	0.42 (0.03)	0.42 (0.03)	0.516	0.41 (0.03)	0.42 (0.03)	0.042
50 kHz-Whole Body Phase Angle	3.1 (2.0)	3.1 (1.7)	3.0 (2.3)	0.720	3.2 (1.9)	3.0 (1.9)	0.075
Skeletal Muscle Index (kg/m^2^)	6.1 (2.2)	6.4 (2.4)	5.8 (1.9)	0.286	5.7 (1.6)	6.3 (2.2)	0.228
Day 3, median (IQR)							
Total Body Water (kg)	30.6 (9.2)	30.4 (9.2)	30.6 (8.4)	0.852	31.0 (9.6)	30.4 (8.8)	0.677
Intracellular Water (kg)	17.9 (5.0)	18.0 (5.7)	17.8 (4.8)	0.650	18.6 (5.7)	17.7 (4.8)	0.517
Extracellular Water (kg)	12.6 (3.5)	12.2 (3.6)	12.8 (3.4)	0.777	13.0 (4.1)	12.3 (3.3)	0.919
Body Fat Mass (kg)	16.7 (14.5)	15.2 (13.9)	18.1 (14.2)	0.265	16.9 (13.8)	16.5 (14.7)	0.721
Soft Lean Mass (kg)	38.9 (10.9)	38.9 (11.9)	38.8 (10.3)	0.785	40.7 (11.9)	38.7 (10.7)	0.604
Skeletal Muscle Mass (kg)	21.3 (6.6)	21.4 (7.4)	21.2 (6.3)	0.656	22.2 (7.3)	21.1 (6.2)	0.523
ECW/TBW	0.41 (0.02)	0.41 (0.02)	0.41 (0.02)	0.505	0.41 (0.03)	0.42 (0.03)	0.006
50 kHz-Whole Body Phase Angle	2.9 (1.7)	3.0 (1.8)	2.7 (1.6)	0.185	3.6 (2.1)	2.6(1.7)	0.002
Skeletal Muscle Index (kg/m^2^)	6.1 (1.7)	6.1 (1.9)	6.0 (1.7)	0.959	5.8 (1.9)	6.1 (1.7)	0.721
Day 8, median (IQR)							
Total Body Water (kg)	29.7 (8.4)	29.8 (8.8)	29.7 (8.5)	0.863	30.2 (10.8)	29.0 (8.1)	0.800
Intracellular Water (kg)	17.6 (4.9)	17.9 (5.1)	17.4 (4.6)	0.628	17.7 (6.4)	17.6 (4.8)	0.718
Extracellular Water (kg)	12.1 (3.9)	12.1 (3.7)	12.4 (4.1)	0.901	12.3 (4.7)	12.2 (3.9)	0.975
Body Fat Mass (kg)	19.5 (13.6)	18.3 (14.4)	20.6 (12.0)	0.175	16.1 (13.9)	20.6 (13.6)	0.379
Soft Lean Mass (kg)	37.7 (11.0)	37.9 (11.2)	37.7 (10.8)	0.828	38.5 (13.9)	36.9 (10.3)	0.797
Skeletal Muscle Mass (kg)	20.9 (6.4)	21.3 (6.6)	20.7 (6.0)	0.646	21.1 (8.3)	20.9 (6.2)	0.712
ECW/TBW	0.41 (0.02)	0.41 (0.02)	0.42 (0.02)	0.309	0.41 (0.02)	0.42 (0.02)	0.028
50 kHz-Whole Body Phase Angle	3.0 (1.7)	2.9 (1.6)	3.0 (1.7)	0.276	3.5 (1.5)	2.8 (1.6)	0.015
Skeletal Muscle Index (kg/m^2^)	5.9 (1.7)	6.1 (1.8)	5.8 (1.8)	0.613	5.9 (2.0)	5.9 (1.7)	0.721
Difference of body composition variable (day 3 value minus day 1 value), median (IQR)							
Total Body Water (kg)	−0.50 (4.85)	−1.05 (4.23)	0.10 (5.40)	0.222	−0.35 (3.88)	−0.50 (5.43)	0.985
Intracellular Water (kg)	−0.50 (2.90)	−0.65 (2.63)	−0.20 (3.00)	0.428	−0.45 (1.98)	−0.50 (3.10)	0.730
Extracellular Water (kg)	0.00 (2.38)	−0.30 (2.20)	0.30 (2.53)	0.218	−0.20 (1.78)	0.05 (2.58)	0.615
Body Fat Mass (kg)	1.50 (4.93)	1.70 (4.43)	1.00 (6.05)	0.234	−1.00 (4.38)	1.85 (5.32)	0.055
Soft Lean Mass (kg)	−0.60 (6.13)	−1.30 (5.93)	−0.25 (6.55)	0.231	−0.55 (4.85)	−0.65 (6.65)	0.897
Skeletal Muscle Mass (kg)	−0.60 (3.80)	−0.80 (3.45)	−0.30 (4.00)	0.405	−0.55 (2.70)	−0.60 (4.10)	0.706
ECW/TBW	0.00 (0.01)	0.00 (0.01)	0.00 (0.01)	0.691	0.00 (0.01)	0.00 (0.01)	0.753
50 kHz-Whole Body Phase Angle	−0.01 (0.88)	−0.10 (0.60)	−0.10 (1.25)	0.860	0.00 (0.88)	−0.20 (0.98)	0.317
Skeletal Muscle Index (kg/m^2^)	−0.01 (1.00)	−0.10 (0.90)	0.20 (1.03)	0.193	0.05 (0.60)	−0.10 (1.00)	0.409

† Comparison analyses between two groups by Mann–Whitney U tests. abbreviations: IQR, interquartile range; ECW/TBW: ratios of extracellular water to total body water.

**Table 5:** Baseline characteristics and primary outcomes of patients, grouped by intervention and nutritional risk.

Four Groups Category	A	B		C	D		
	Intervention with High Risk (*n* = 49)	Intervention with Low Risk (*n* = 20)	*p* †	Non-Intervention with High Risk (*n* = 44)	Non-Intervention with Low Risk (*n* = 19)	*p* †	*p* *
Demographic characteristics, mean (SD) or median (IQR)							
Age (years)	76.1 (10.8)	64.0 (10.5)	0.000	71.9 (11.8)	63.0 (9.7)	0.023	0.000
Gender(female), *n* (%)	22 (44.9)	7 (35.0)	0.453	21 (47.7)	7 (38.9)	0.529	0.779
Body weight (kg)	58.1 (13.2)	65.2 (16.4)	0.406	62.6 (16.0)	57.1 (12.1)	1.000	0.150
BMI (kg/m2)	22.9 (4.8)	24.4 (6.2)	1.000	24.1 (6.4)	21.4 (4.2)	0.451	0.236
APACHE II	24.6 (5.2)	15.8 (5.5)	0.000	24.8 (6.3)	13.8 (3.6)	0.000	0.000
PSI score	144.7 (42.9)	109.3 (25.0)	0.000	144.8 (47.1)	107.2 (38.5)	0.008	0.000
CURB-65 score	2.0 (1.0)	1.0 (2.0)	0.000	2.0 (2.0)	1.0 (1.0)	0.008	0.000
Charlson comorbidity index	6.0 (3.0)	4.0 (3.0)	0.001	6.0 (4.0)	4.0 (3.0)	0.030	0.002
mNUTRIC score	6.0 (2.0)	3.5 (1.0)	0.000	6.0 (2.0)	3.0 (1.0)	0.000	0.000
Site of suspected infection, *n* (%)							
Lung	43 (87.8)	13 (65.0)	0.029	39 (88.6)	14 (73.7)	0.139	0.062
Intra-abdomen	3 (6.1)	1 (5.0)	0.857	2 (4.5)	0 (0.0)	0.349	0.756
UTI	38 (77.6)	15 (75.0)	0.821	29 (65.9)	11 (59.7)	0.547	0.357
Bacteremia	26 (53.1)	14 (70.0)	0.199	22 (50.0)	9 (47.4)	0.849	0.445
Others	0 (0.0)	1 (5.0)	0.118	2 (4.5)	2 (10.5)	0.375	0.220
Length of stay, median (IQR)							
ICU Days	10.7 (8.2)	7.3 (10.7)	0.224	9.0 (8.4)	6.6 (4.7)	0.065	0.054
Hospital days	29.4 (34.3)	28.4 (33.9)	0.526	30.8 (31.2)	18.2 (14.5)	0.245	0.437
Total MV days	14.0 (23.2)	9.3 (31.8)	0.098	10.8 (25.6)	7.6 (14.9)	0.178	0.036
mortality, *n* (%)							
ICU Mortality,	6 (12.2)	2 (10.0)	0.793	5 (11.4)	2 (11.1)	0.977	0.995
Hospital Mortality	10 (20.4)	4 (20.0)	0.970	10 (22.7)	3 (16.7)	0.598	0.961
7-day Mortality	1 (2.0)	0 (0.0)	0.523	3 (6.8)	1 (5.6)	0.855	0.489
28-day Mortality	5 (10.2)	2 (10.0)	0.980	6 (13.6)	3 (16.7)	0.760	0.876

† Comparison analyses between two groups by Mann–Whitney U tests or chi-squared tests for categorical variables. *p* *: Comparison analyses among three groups using one-way analysis of variance (ANOVA), with Kruskal–Wallis as a non-parametric alternative to ANOVA for non-normally distributed continuous variables or chi-square tests for categorical variables. Abbreviations: SD, standard deviation; IQR, interquartile range; BMI, body mass index; APACHE, acute physiology and chronic health evaluation; PSI, pneumonia severity index, CURB-65: Confusion, blood urea > 42.8 mg/dL, respiratory rate > 30/min, blood pressure < 90/60 mmHg, age > 65; mNUTRIC, modified nutrition risk in critically ill.

**Table 6:** Items of statistically significant differences between groups by intervention and nutritional risk.

	Intervention > Control	Intervention < Control	High-Risk > Low-Risk	Low-Risk > High-Risk
baseline			Age, APACH II, PSI, CURB 65, Charlson comorbidity index, Modified NUTRIC score, Lung infection, HT, Diabetes mellitus, CKD	
outcomes			ICU days Total MV days	Amount of caloric and protein intake (day 1, 2, 3, 4, 5, 6, 8; day 1, 2, 3, 4, 5, 6, 8) Percentage of meeting caloric and protein intake goals (day 1, 2, 3, 4, 5; day 4, 5, 6, 8)
Serial SOFA	Day 8 SOFA score		Day 1, 3, and 8 SOFA score	ΔSOFA
Body composition variable			Day 1, 3, and 8 ECW/TBW	Day 3 and 8, 50 kHz-Whole Body Phase Angle

**Table 7:** Items of Statistically Significant Differences Between Groups by Intervention and Nutritional Risk.

	A > B	B > A	C > D	C < D
baseline	Age, APACH II, PSI, CURB 65, Charlson comorbidity index, Modified NUTRIC score, Lung infection		Age, APACH II, PSI, CURB 65, Charlson comorbidity index, Modified NUTRIC score, Lung infection	Amount of caloric intake (day 5)
outcomes		Amount of caloric and protein intake (day 1, 4, 5, 8; day 1, 4, 5, 6, 7, 8) Percentage of meeting caloric and protein intake goals (day 1, 3, 4, 5; day 3, 4, 5, 6, 8)		
Serial SOFA	Day 1 and 8 SOFA score		Day 1, 3, and 8 SOFA score	ΔSOFA
Body composition variable	ΔBody Fat Mass		Day 3 ECW/TBW	Day 3 50 kHz-Whole Body Phase Angle Δ50 kHz-Whole Body Phase Angle

## Data Availability

The data presented in this study are available on request from the corresponding author. The data are not publicly available due to patients’ privacy.

## Supplementary Materials

The following supporting information can be downloaded at: https://www.mdpi.com/article/10.3390/nu15173814/s1, Figure S1: Survival Curves for Control and Intervention Groups; Figure S2: Survival Curves Between Low-Risk and High-Risk Groups; Figure S3: Survival Curves Among the Four Groups; Table S1: Amounts of Caloric and Protein Intake of Patients, Grouped by Intervention or Nutritional Risk; Table S2: Percentage of Patients Meeting Caloric or Protein Goals, Grouped by Intervention or Nutritional Risk; Table S3: Pairwise Comparison Among Groups; Table S4: Amounts of Caloric and Protein Intake Among the Four Groups; Table S5: Percentage of Patients Meeting Caloric or Protein Goals Among the Four Groups; Table S6: Serial Severity Scores Among the Four Groups; Table S7: Serial Body Composition Variables Among the Four Groups.

## References

[1] Li A., Ling L., Qin H., Arabi Y.M., Myatra S.N., Egi M., Kim J.H., Mat Nor M.B., Son D.N., Fang W.F. (2022). Epidemiology, Management, and Outcomes of Sepsis in ICUs among Countries of Differing National Wealth across Asia. Am. J. Respir. Crit. Care Med..

[2] Manga G., Calin G.A., Manuc M., Droc G., Tudor S. (2018). New Definitions of Sepsis and the Quest for Specific Biomarkers. Are the miRNAs the Answer?. Chirurgia.

[3] Singer M., Deutschman C.S., Seymour C.W., Shankar-Hari M., Annane D., Bauer M., Bellomo R., Bernard G.R., Chiche J.D., Coopersmith C.M. (2016). The Third International Consensus Definitions for Sepsis and Septic Shock (Sepsis-3). JAMA.

[4] Fang W.F., Douglas I.S., Chen Y.M., Lin C.Y., Kao H.C., Fang Y.T., Huang C.H., Chang Y.T., Huang K.T., Wang Y.H. (2017). Development and validation of immune dysfunction score to predict 28-day mortality of sepsis patients. PLoS ONE.

[5] Fang W.F., Chen Y.M., Lin C.Y., Huang K.T., Kao H.C., Fang Y.T., Huang C.H., Chang Y.T., Wang Y.H., Wang C.C. (2017). Immune profiles and clinical outcomes between sepsis patients with or without active cancer requiring admission to intensive care units. PLoS ONE.

[6] Hung K.Y., Chen Y.M., Wang C.C., Wang Y.H., Lin C.Y., Chang Y.T., Huang K.T., Lin M.C., Fang W.F. (2019). Insufficient Nutrition and Mortality Risk in Septic Patients Admitted to ICU with a Focus on Immune Dysfunction. Nutrients.

[7] Waitzberg D.L., Caiaffa W.T., Correia M.I. (2001). Hospital malnutrition: The Brazilian national survey (IBRANUTRI): A study of 4000 patients. Nutrition.

[8] Correia M.I., Campos A.C., Study E.C. (2003). Prevalence of hospital malnutrition in Latin America: The multicenter ELAN study. Nutrition.

[9] Kamath S.K., Lawler M., Smith A.E., Kalat T., Olson R. (1986). Hospital malnutrition: A 33-hospital screening study. J. Am. Diet. Assoc..

[10] O’Flynn J., Peake H., Hickson M., Foster D., Frost G. (2005). The prevalence of malnutrition in hospitals can be reduced: Results from three consecutive cross-sectional studies. Clin. Nutr..

[11] Artinian V., Krayem H., DiGiovine B. (2006). Effects of early enteral feeding on the outcome of critically ill mechanically ventilated medical patients. Chest.

[12] Marik P.E., Hooper M.H. (2016). Normocaloric versus hypocaloric feeding on the outcomes of ICU patients: A systematic review and meta-analysis. Intensive Care Med..

[13] Arabi Y.M., Aldawood A.S., Haddad S.H., Al-Dorzi H.M., Tamim H.M., Jones G., Mehta S., McIntyre L., Solaiman O., Sakkijha M.H. (2015). Permissive Underfeeding or Standard Enteral Feeding in Critically Ill Adults. N. Engl. J. Med..

[14] Arabi Y.M., Aldawood A.S., Al-Dorzi H.M., Tamim H.M., Haddad S.H., Jones G., McIntyre L., Solaiman O., Sakkijha M.H., Sadat M. (2017). Permissive Underfeeding or Standard Enteral Feeding in High- and Low-Nutritional-Risk Critically Ill Adults. Post Hoc Analysis of the PermiT Trial. Am. J. Respir. Crit. Care Med..

[15] Paixão E.M.S., Gonzalez M.C., Nakano E.Y., Ito M.K., Pizato N. (2021). Weight loss, phase angle, and survival in cancer patients undergoing radiotherapy: A prospective study with 10-year follow-up. Eur. J. Clin. Nutr..

[16] Arab A., Karimi E., Vingrys K., Shirani F. (2021). Is phase angle a valuable prognostic tool in cancer patients’ survival? A systematic review and meta-analysis of available literature. Clin. Nutr..

[17] Wischmeyer P.E., Bear D.E., Berger M.M., De Waele E., Gunst J., McClave S.A., Prado C.M., Puthucheary Z., Ridley E.J., Van den Berghe G. (2023). Personalized nutrition therapy in critical care: 10 expert recommendations. Crit. Care.

[18] McClave S.A., Taylor B.E., Martindale R.G., Warren M.M., Johnson D.R., Braunschweig C., McCarthy M.S., Davanos E., Rice T.W., Cresci G.A. (2016). Guidelines for the Provision and Assessment of Nutrition Support Therapy in the Adult Critically Ill Patient: Society of Critical Care Medicine (SCCM) and American Society for Parenteral and Enteral Nutrition (A.S.P.E.N.). J. Parenter. Enter. Nutr..

[19] Arabi Y.M., Phua J., Koh Y., Du B., Faruq M.O., Nishimura M., Fang W.F., Gomersall C., Al Rahma H.N., Tamim H. (2016). Structure, Organization, and Delivery of Critical Care in Asian ICUs. Crit. Care Med..

[20] See K.C., Zhao M.Y., Nakataki E., Chittawatanarat K., Fang W.F., Faruq M.O., Wahjuprajitno B., Arabi Y.M., Wong W.T., Divatia J.V. (2018). Professional burnout among physicians and nurses in Asian intensive care units: A multinational survey. Intensive Care Med..

[21] Tseng C.C., Tu C.Y., Chen C.H., Wang Y.T., Chen W.C., Fu P.K., Chen C.M., Lai C.C., Kuo L.K., Ku S.C. (2021). Significance of the Modified NUTRIC Score for Predicting Clinical Outcomes in Patients with Severe Community-Acquired Pneumonia. Nutrients.

[22] Lin C.Y., Wang Y.H., Chen Y.M., Hung K.Y., Chang Y.C., Fang Y.T., Chang Y.T., Chen H.C., Huang K.T., Chang H.C. (2021). Dynamic monitoring of kidney injury status over 3 days in the intensive care unit as a sepsis phenotype associated with hospital mortality and hyperinflammation. Biomed. J..

[23] Huang M., Han M., Song Z., Kuang L. (2023). Stress ulcer prophylaxis in critically ill adult patients with sepsis at risk of gastrointestinal bleeding: A retrospective cohort study. Intern. Med. J..

[24] Karakike E., Kyriazopoulou E., Tsangaris I., Routsi C., Vincent J.L., Giamarellos-Bourboulis E.J. (2019). The early change of SOFA score as a prognostic marker of 28-day sepsis mortality: Analysis through a derivation and a validation cohort. Crit. Care.

[25] Chao W.C., Tseng C.H., Chien Y.C., Sheu C.C., Tsai M.J., Fang W.F., Chen Y.M., Kao K.C., Hu H.C., Perng W.C. (2018). Association of day 4 cumulative fluid balance with mortality in critically ill patients with influenza: A multicenter retrospective cohort study in Taiwan. PLoS ONE.

[26] Ko S.J., Cho J., Choi S.M., Park Y.S., Lee C.H., Lee S.M., Yoo C.G., Kim Y.W., Lee J. (2021). Phase Angle and Frailty Are Important Prognostic Factors in Critically Ill Medical Patients: A Prospective Cohort Study. J. Nutr. Health Aging.

[27] Puthucheary Z.A., Rawal J., McPhail M., Connolly B., Ratnayake G., Chan P., Hopkinson N.S., Phadke R., Dew T., Sidhu P.S. (2013). Acute skeletal muscle wasting in critical illness. JAMA.

[28] Casey P., Alasmar M., McLaughlin J., Ang Y., McPhee J., Heire P., Sultan J. (2022). The current use of ultrasound to measure skeletal muscle and its ability to predict clinical outcomes: A systematic review. J. Cachexia Sarcopenia Muscle.

[29] Phua J., Faruq M.O., Kulkarni A.P., Redjeki I.S., Detleuxay K., Mendsaikhan N., Sann K.K., Shrestha B.R., Hashmi M., Palo J.E.M. (2020). Critical Care Bed Capacity in Asian Countries and Regions. Crit. Care Med..

