# Supplementary material for: Using Body Composition Analysis for Improved Nutritional Intervention in Septic Patients: A Prospective Interventional Study
Source: Nutrients. 2023 Aug 31;15(17):3814. doi: 10.3390/nu15173814 (PMC10489810; doi:10.3390/nu15173814)
Supplement: Supplementary file 1 [file nutrients-15-03814-s001.zip › nutrients-2551406-supplementary.pdf]

Supplemental Material

Title: Using Body Composition Analysis for Improved Nutritional Intervention in Septic Patients: A Prospective Interventional Study

Kai-Yin Hung, Tzu-Hsiu Chen, Ya-Fen Lee, Wen-Feng Fang

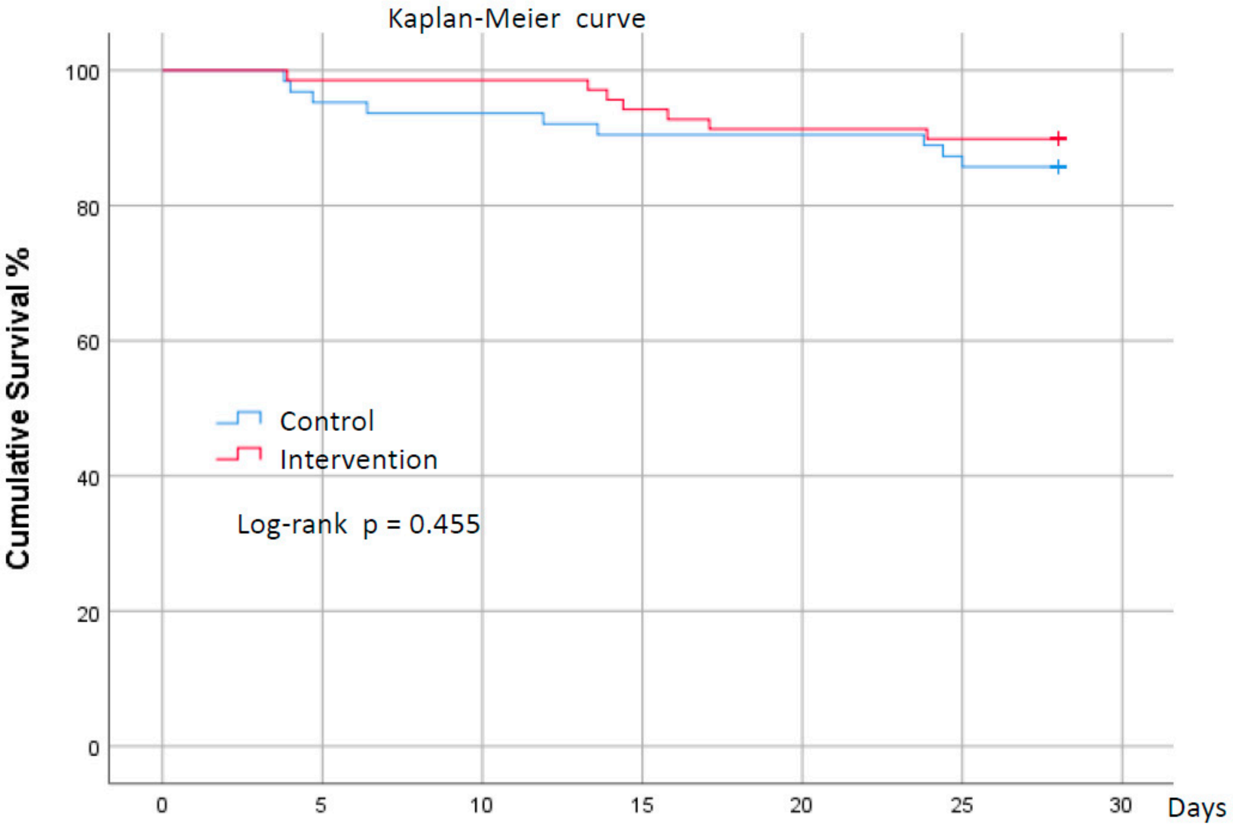

Figure S1: Survival Curves for Control and Intervention Groups

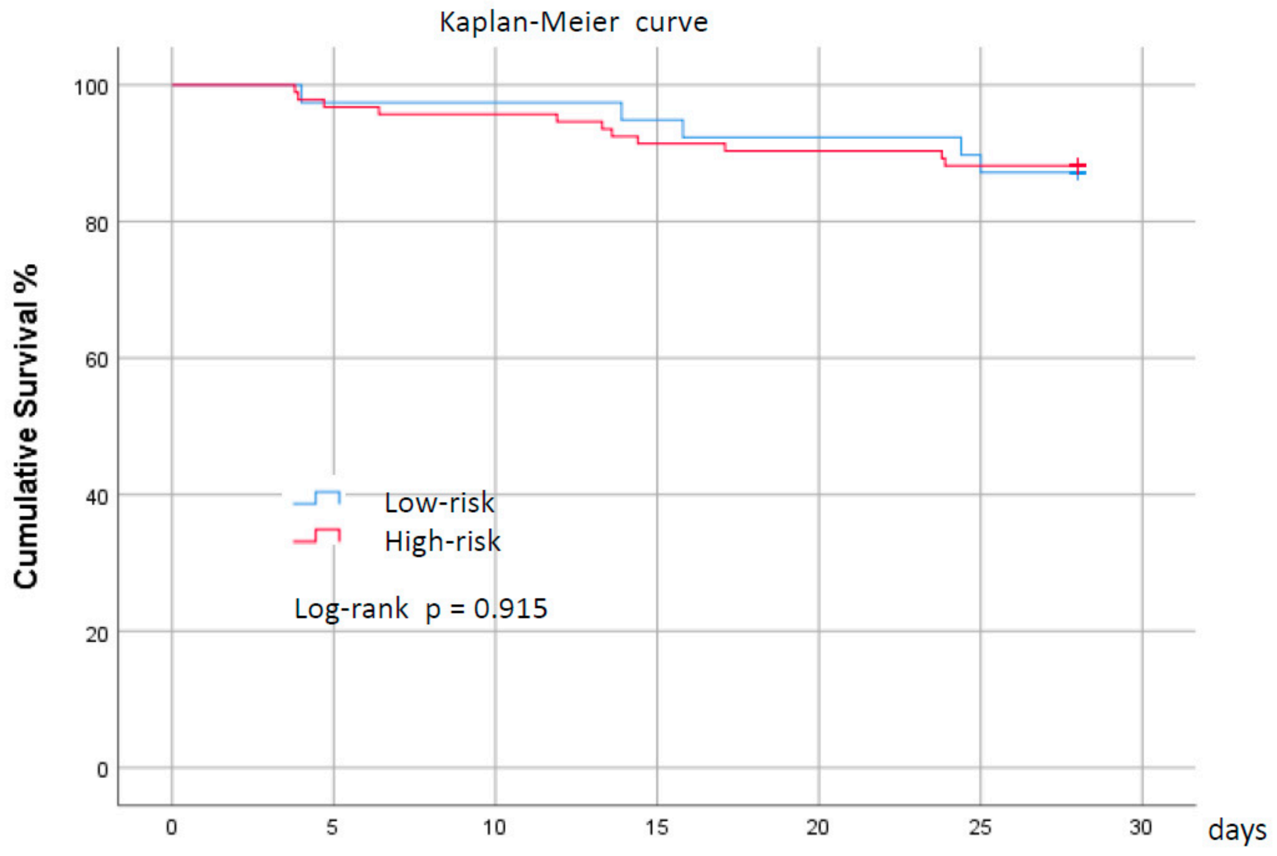

Figure S2: Survival Curves Between Low-Risk and High-Risk Groups

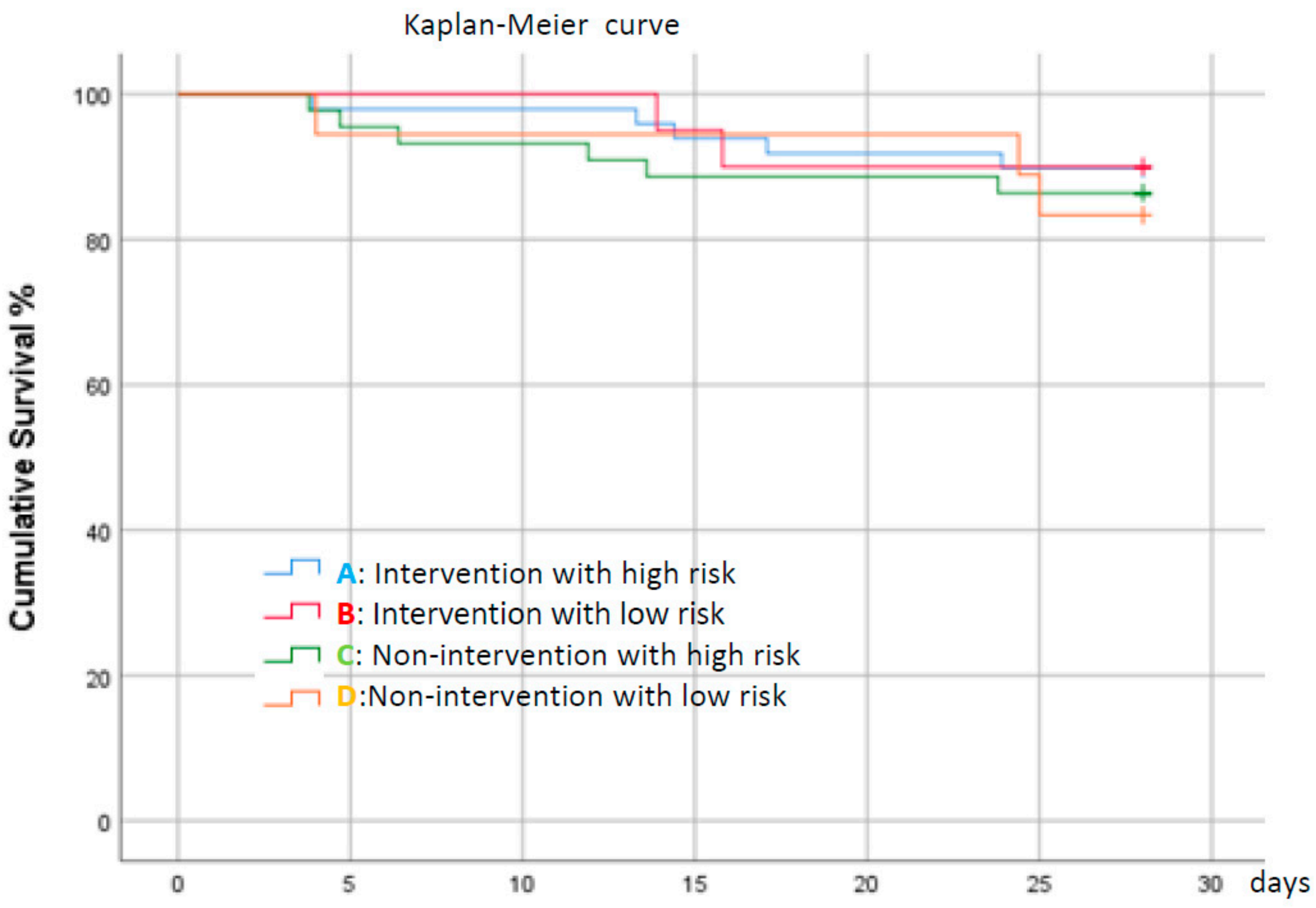

Figure S3: Survival Curves Among the Four Groups

Table S1: Amounts of Caloric and Protein Intake of Patients, Grouped by Intervention or Nutritional Risk

	All	control	intervention		Low-risk	High-risk	
	N=132	N=63	N=69	p†	N=39	N=93	p†
**Caloric intake**, kcal/kg, median (IQR)							
Day 1	11.9 (12.6)	11.7 (12.2)	11.9 (12.4)	0.594	19.5 (13.4)	9.9 (10.3)	0.001
Day 2	14.4 (11.2)	14.2 (12.4)	15.2 (10.1)	0.622	17.9 (13.3)	13.7 (9.8)	0.027
Day 3	14.6 (9.4)	14.3 (8.9)	14.7 (10.7)	0.619	19.5 (10.2)	13.9 (7.9)	0.008
Day 4	16.5 (8.2)	15.7 (8.8)	16.5 (8.2)	0.942	19.8 (6.8)	15.1 (8.3)	0.001
Day 5	16.7 (9.1)	15.8 (9.1)	16.8 (9.0)	0.864	20.3 (6.6)	15.0 (9.0)	0.000
Day 6	16.5 (8.8)	16.0 (8.7)	16.8 (8.8)	0.493	19.6 (9.4)	15.4 (8.3)	0.018
Day 7	16.8 (7.6)	16.7 (8.0)	16.8 (7.3)	0.728	18.5 (9.6)	16.0 (6.9)	0.127
Day 8	16.6 (9.0)	16.4 (9.1)	16.6 (8.5)	0.880	21.7 (9.0)	15.6 (8.3)	0.004
**Protein intake**, g/kg, median (IQR)							
Day 1	0.6 (0.6)	0.5 (0.8)	0.5 (0.8)	0.914	0.7 (0.7)	0.4 (0.7)	0.014
Day 2	0.6 (0.6)	0.7 (0.7)	0.6 (0.6)	0.204	0.8 (0.6)	0.6 (0.6)	0.021
Day 3	0.7 (0.5)	0.7 (0.5)	0.6 (0.5)	0.337	0.8 (0.6)	0.6 (0.5)	0.029
Day 4	0.8 (0.5)	0.8 (0.5)	0.8 (0.5)	0.897	0.9 (0.5)	0.7 (0.5)	0.001
Day 5	0.8 (0.5)	0.8 (0.5)	0.8 (0.5)	0.974	1.0 (0.4)	0.7 (0.5)	0.001
Day 6	0.8 (0.5)	0.8 (0.5)	0.8 (0.5)	0.837	1.0 (0.5)	0.8 (0.5)	0.011
Day 7	0.8 (0.5)	0.8 (0.5)	0.8 (0.4)	0.551	0.9 (0.5)	0.8 (0.5)	0.182
Day 8	0.8 (0.5)	0.9 (0.6)	0.8 (0.5)	0.663	1.1 (0.5)	0.8 (0.5)	0.020

† Comparison analyses between two groups by Mann-Whitney U tests. abbreviations: IQR, interquartile range

Table S2: Percentage of Patients Meeting Caloric or Protein Goals, Grouped by Intervention or Nutritional Risk

	All	control	intervention		Low-risk	High-risk	
	N=132	N=63	N=69	p†	N=39	N=93	p†
**Meet caloric goal**, n (%)							
Day 1	25 (19.8)	11 (18.3)	14 (21.2)	0.687	15 (45.5)	10 (10.8)	0.000
Day 2	26 (20.5)	15 (25.0)	11 (16.4)	0.233	12 (35.3)	14 (15.1)	0.024
Day 3	29 (24.2)	13 (23.6)	16 (24.6)	0.901	14 (46.7)	15 (16.7)	0.002
Day 4	34 (29.6)	17 (33.3)	17 (26.6)	0.431	14 (50.0)	20 (23.0)	0.007
Day 5	36 (32.7)	16 (32.0)	20 (33.3)	0.883	16 (57.1)	20 (24.4)	0.002
Day 6	35 (33.0)	14 (29.2)	21 (36.2)	0.445	12 (48.0)	23 (28.4)	0.070
Day 7	25 (26.9)	12 (29.3)	13 (25.0)	0.647	7 (36.8)	18 (24.3)	0.275
Day 8	30 (34.1)	16 (40.0)	14 (29.2)	0.288	9 (52.9)	21 (29.6)	0.070
**Meet protein goal**, n (%)							
Day 1	8 (6.3)	5 (8.3)	3 (4.5)	0.386	2 (6.1)	6 (6.5)	0.482
Day 2	9 (7.5)	5 (8.3)	4 (6.0)	0.606	4 (11.8)	5 (5.4)	0.454
Day 3	9 (7.5)	5 (9.1)	4 (6.2)	0.544	5 (16.7)	4 (4.4)	0.082
Day 4	10 (8.7)	4 (7.8)	6 (9.4)	0.773	7 (25.0)	3 (3.4)	0.000
Day 5	11 (10.0)	4 (8.0)	7 (11.7)	0.525	7 (25.0)	4 (4.9)	0.002
Day 6	13 (12.3)	8 (16.7)	5 (8.6)	0.211	6 (24.0)	7 (8.6)	0.042
Day 7	9 (9.7)	5 (12.2)	4 (7.7)	0.468	3 (15.8)	6 (8.1)	0.315
Day 8	13 (14.8)	6 (15.0)	7 (14.6)	0.957	6 (35.3)	7 (9.9)	0.008

† Comparison analyses between two groups by chi-squared tests for categorical variables.

Table S3: Pairwise Comparison Among Groups

Pairwise comparison									
**Group**		A		B		C		D	
		Chi-square	significant	Chi-square	significant	Chi-square	significant	Chi-square	significant
Log Rank (Mantel-Cox)	A			0.001	0.980	0.303	0.582	0.349	0.555
	B	0.001	0.980			0.203	0.653		0.612
	C	0.303	0.582	0.203	0.653				0.865
	D	0.349	0.555	0.257	0.612	0.029	0.865		

Table S4: Amounts of Caloric and Protein Intake Among the Four Groups

Four groups category	A	B		C	D		
	Intervention with high risk (n = 49)	Intervention with low risk (n = 20)	p†	Non-intervention with high risk (n = 44)	Non-intervention with low risk (n = 19)	p†	p*
**Caloric intake**, kcal/kg, median (IQR)							
Day 1	10.3 (12.4)	19. 8(12.7)	0.006	9.8 (9.6)	17.9 (16.8)	0.091	0.013
Day 2	14.0 (10.1)	15.7 (11.4)	0.289	13.5 (9.7)	19.3 (16.4)	0.064	0.128
Day 3	13.7 (8.8)	20.2 (10.8)	0.068	14.1 (7.7)	19.2 (11.5)	0.066	0.066
Day 4	16.0 (7.3)	20.2 (8.4)	0.009	14.1 (9.3)	19.5 (6.3)	0.110	0.017
Day 5	16.1 (9.2)	20.3 (7.3)	0.010	13.3 (9.2)	20.6 (6.7)	0.009	0.004
Day 6	16. 3(8.4)	20.2 (10.4)	0.148	14.5 (8.3)	19.6 (8.5)	0.055	0.097
Day 7	15.3 (7.6)	18.5 (7.4)	0.065	16.7 (7.8)	17.5 (11.2)	0.895	0.287
Day 8	15.9 (6.8)	19.6 (11.0)	0.012	15.3 (8.5)	21.7 (6.0)	0.194	0.039
**Protein intake**, g/kg, median (IQR)							
Day 1	0.4 (0.7)	0.7 (0.5)	0.022	0.4 (0.7)	0.7 (0.9)	0.262	0.090
Day 2	0.6 (0.6)	0.8 (0.5)	0.069	0.6 (0.7)	0.9 (0.7)	0.150	0.073
Day 3	0.6 (0.5)	0.8 (0.7)	0.127	0.7 (0.5)	0.8 (0.6)	0.142	0.130
Day 4	0.7 (0.4)	0.9 (0.6)	0.002	0.8 (0.6)	0.9 (0.5)	0.173	0.009
Day 5	0.7 (0.5)	1.0 (0.5)	0.004	0.8 (0.6)	0.9 (0.4)	0.083	0.009
Day 6	0.7 (0.5)	1.0 (0.5)	0.031	0.8 (0.5)	0.9 (0.5)	0.178	0.083
Day 7	0.7 (0.4)	1.0 (0.4)	0.034	0.9 (0.6)	0.7 (0.5)	0.705	0.187

Day 8	0.8 (0.4)	1.2 (0.6)	0.011	0.9 (0.7)	1.0 (0.3)	0.606	0.084

† Comparison analyses between two groups by Mann-Whitney U tests.

p *: Comparison analyses among three groups using one-way analysis of variance (ANOVA), with Kruskal-Wallis as a non-parametric alternative to ANOVA for non-normally distributed continuous variables.

abbreviations: IQR, interquartile range

Table S5: Percentage of Patients Meeting Caloric or Protein Goals Among the Four Groups

Four groups category	A	B		C	D		
	Intervention with high risk (n = 49)	Intervention with low risk (n = 20)	p†	Non-intervention with high risk (n = 44)	Non-intervention with low risk (n = 19)	p†	p*
**Meet caloric goal**, n (%)							
Day 1	6 (12.2)	8 (47.1)	0.003	4 (9.1)	6 (40.0)	0.006	0.001
Day 2	6 (12.2)	5 (27.8)	0.131	8 (18.2)	6 (40.0)	0.089	0.096
Day 3	7 (14.3)	9 (56.3)	0.001	8 (19.5)	4 (30.8)	0.399	0.006
Day 4	9 (18.4)	8 (53.3)	0.008	11 (28.9)	6 (46.2)	0.261	0.034
Day 5	11 (24.4)	9 (60.0)	0.012	9 (24.3)	7 (53.8)	0.052	0.017
Day 6	14 (31.1)	7 (53.8)	0.136	9 (25.0)	5 (41.7)	0.276	0.257
Day 7	9 (22.0)	4 (36.4)	0.332	9 (27.3)	3 (37.5)	0.573	0.692
Day 8	9 (27.3)	5 (50.0)	0.107	12 (36.4)	4 (57.1)	0.314	0.200
**Meet protein goal**, n (%)							
Day 1	2 (4.1)	1 (5.9)	0.761	4 (9.1)	0 (0.0)	0.230	0.551
Day 2	2 (4.1)	2 (11.1)	0.285	3 (6.8)	1 (6.7)	0.984	0.772
Day 3	1 (2.0)	3 (18.8)	0.017	3 (7.3)	1 (7.7)	0.964	0.145
Day 4	1 (2.0)	5 (33.3)	0.000	2 (5.3)	2 (15.4)	0.246	0.002
Day 5	2 (4.4)	5 (33.3)	0.003	2 (5.4)	2 (15.4)	0.259	0.008

Day 6	2 (4.4)	3 (23.1)	0.037	5 (13.9)	3 (25.0)	0.376	0.121
Day 7	2 (4.9)	2 (18.2)	0.145	4 (12.1)	1 (12.5)	0.977	0.519
Day 8	2 (5.3)	5 (50.0)	0.000	5 (15.2)	1 (14.3)	0.954	0.006

† Comparison analyses between two groups by chi-squared tests for categorical variables.

p *: Comparison analyses among three groups using chi-square tests for categorical variables.

Table S6: Serial Severity Scores Among the Four Groups

Four groups category	A	B		C	D		
	Intervention with high risk (n = 49)	Intervention with low risk (n = 20)	p†	Non-intervention with high risk (n = 44)	Non-intervention with low risk (n = 19)	p†	p*
**Serial severity scores**, median (IQR)							
Day 1 SOFA	8 (4)	4.5 (6)	0.000	8 (5)	5 (4)	0.000	0.000
Day 3 SOFA	6 (4)	6.0 (4)	0.134	6 (4)	4 (3)	0.016	0.039
Day 8 SOFA	6 (5)	4.5 (5)	0.016	6 (5)	3 (4)	0.012	0.000
**Difference of severity score** (day 3 value minus day 1 value), median (IQR)							
△ SOFA	-2 (3)	-1.5 (7)	0.369	-3 (4)	-2 (4)	0.021	0.014

† Comparison analyses between two groups by Mann-Whitney U tests.

p *: Comparison analyses among three groups using one-way analysis of variance (ANOVA), with Kruskal-Wallis as a non-parametric alternative to ANOVA for non-normally distributed continuous variables.

abbreviations: IQR, interquartile range; SOFA, sequential organ failure assessment

Table S7: Serial Body Composition Variables Among the Four Groups

Four groups category	A	B		C	D		
	Intervention with high risk (n = 49)	Intervention with low risk (n = 20)	p†	Non-intervention with high risk (n = 44)	Non-intervention with low risk (n = 19)	p†	p*
**Day 1**, median (IQR)							
Total Body Water (kg)	28.7 (8.8)	30.2 (8.2)	0.287	30.9 (8.2)	29.5 (7.9)	0.445	0.231
Intracellular Water (kg)	17.2 (5.5)	17.8 (4.3)	0.354	19.2 (5.4)	17.7 (5.1)	0.397	0.233
Extracellular Water (kg)	11.6 (3.4)	12.3 (3.5)	0.202	12.8 (4.7)	11.9 (3.6)	0.432	0.192
Body Fat Mass (kg)	18.8 (15.1)	19.7 (17.5)	0.137	17.7 (12.9)	11.9 (8.5)	0.159	0.077
Soft Lean Mass (kg)	36.5 (11.7)	38.3 (10.1)	0.299	39.56 (10.5)	39.8 (11.9)	0.520	0.209
Skeletal Muscle Mass (kg)	20.6 (7.4)	21.2 (5.6)	0.383	23.0 (7.1)	22.6 (8.1)	0.429	0.234
ECW/TBW	0.42 (0.03)	0.41 (0.03)	0.255	0.42 (0.03)	0.40 (0.03)	0.073	0.217
50kHz-Whole Body Phase Angle	3.0 (2.4)	3.2 (2.0)	0.389	3.0 (1.8)	3.1 (1.8)	0.107	0.367
Skeletal Muscle Index (kg/m2)	5.9 (2.1)	5.7 (1.5)	0.910	6.5 (2.5)	6.0 (2.4)	0.167	0.309
**Day 3**, median (IQR)							
Total Body Water (kg)	30.3 (8.6)	32.6 (8.4)	0.169	30.5 (9.8)	29.3 (8.5)	0.418	0.519
Intracellular Water (kg)	17.7 (4.1)	19.3 (4.7)	0.157	18.1 (5.3)	17.6 (6.6)	0.702	0.531
Extracellular Water (kg)	12.6 (3.3)	13.4 (4.0)	0.239	12.5 (3.8)	11.7 (3.9)	0.202	0.455
Body Fat Mass (kg)	17.0 (17.2)	19.0 (14.6)	0.252	15.9 (14.5)	12.6 (12.4)	0.472	0.344
Soft Lean Mass (kg)	38.5 (10.7)	41.5 (10.4)	0.153	39.0 (11.9)	37.4 (11.0)	0.489	0.511

Skeletal Muscle Mass (kg)	21.1 (5.4)	23.1 (6.1)	0.165	21.6 (7.0)	20.9 (8.5)	0.695	0.545
ECW/TBW	0.42 (0.03)	0.41 (0.03)	0.066	0.42 (0.02)	0.41 (0.03)	0.039	0.052
50kHz-Whole Body Phase Angle	2.5 (1.9)	3.4 (2.1)	0.062	2.7 (1.6)	3.7 (2.2)	0.012	0.010
Skeletal Muscle Index (kg/m2)	6.1 (1.7)	5.9 (1.9)	0.716	6.1 (1.8)	5.6 (2.2)	0.377	0.816
**Day 8**, median (IQR)							
Total Body Water (kg)	28.4 (8.3)	32.2 (10.2)	0.210	31.0 (8.9)	28.8 (8.4)	0.305	0.535
Intracellular Water (kg)	17.1 (4.5)	18.6 (5.4)	0.164	18.3 (4.7)	17.1 (5.2)	0.340	0.422
Extracellular Water (kg)	11.9 (4.1)	13.6 (4.7)	0.282	12.6 (3.6)	11.5 (3.4)	0.242	0.555
Body Fat Mass (kg)	20.5 (13.0)	19.4 (18.6)	0.477	20.6 (14.1)	11.5 (14.6)	0.106	0.182
Soft Lean Mass (kg)	36.3 (10.3)	40.7 (12.7)	0.199	39.4 (11.0)	37.0 (11.4)	0.299	0.522
Skeletal Muscle Mass (kg)	20.2 (5.9)	22.2 (7.0)	0.164	21.8 (6.1)	20.3 (7.4)	0.347	0.431
ECW/TBW	0.42 (0.02)	0.41 (0.02)	0.206	0.42 (0.02)	0.41 (0.03)	0.133	0.179
50kHz-Whole Body Phase Angle	2.8 (1.9)	3.4 (1.2)	0.161	2.7 (1.5)	3.8 (2.7)	0.065	0.101
Skeletal Muscle Index (kg/m2)	5.7 (1.7)	6.2 (1.5)	0.141	6.1 (1.8)	5.5 (2.0)	0.360	0.384
**Difference of body composition variable** (day 3 value minus day 1 value), median (IQR)							
Total Body Water (kg)	-0.25 (5.60)	0.45 (6.90)	0.686	-1.10 (5.33)	1.00 (2.15)	0.784	0.612
Intracellular Water (kg)	-0.35 (2.98)	-0.10 (3.55)	0.489	-0.65 (3.18)	-0.65 (1.38)	0.905	0.773
Extracellular Water (kg)	0.05 (2.58)	0.55 (3.28)	0.874	-0.10 (2.55)	-0.30 (0.98)	0.519	0.523
Body Fat Mass (kg)	1.65 (6.08)	-1.95 (6.23)	0.028	2.20 (4.75)	1.20 (3.20)	0.452	0.223
Soft Lean Mass (kg)	-0.50 (6.68)	0.45 (8.55)	0.574	-1.30 (6.75)	-2.00 (3.05)	0.841	0.995
Skeletal Muscle Mass (kg)	-0.45 (3.95)	-0.15 (4.60)	0.471	-0.80 (4.18)	-0.70 (1.95)	0.890	0.987
ECW/TBW	0.00 (0.01)	0.01 (0.01)	0.202	0.00 (0.01)	0.00 (0.01)	0.309	0.838
50kHz-Whole Body Phase Angle	-0.10 (1.03)	-0.20 (1.40)	0.635	-0.20 (0.78)	0.00 (0.95)	0.026	0.881

Skeletal Muscle Index (kg/m2)	-0.05 (1.00)	0.20 (1.18)	0.348	-0.35 (1.13)	-0.10 (0.65)	0.707	0.751

† Comparison analyses between two groups by Mann-Whitney U tests.

p *: Comparison analyses among three groups using one-way analysis of variance (ANOVA), with Kruskal-Wallis as a non-parametric alternative to ANOVA for non-normally distributed continuous variables.

abbreviations: IQR, interquartile range; ECW/TBW: ratios of extracellular water to total body water
